# Minimally invasive treatment of female stress urinary incontinence with the adjustable single-incision sling system (AJUST ™) in an elderly and overweight population

**DOI:** 10.1590/S1677-5538.IBJU.2015.0751

**Published:** 2017

**Authors:** Ralf Anding, Manuel Schoen, Ruth Kirschner-Hermanns, Christian Fisang, Stefan C. Müller, Stefan Latz

**Affiliations:** 1 Department of Urology and Neuro-Urology, University Hospital Bonn, Germany;; 2 Department of Urology, Klinikum Ibbenbueren GmbH, Germany

**Keywords:** Body Mass Index, Obesity, Suburethral Slings, Urinary Incontinence, Stress

## Abstract

**Introduction:**

The prevalence of urinary incontinence is increasing. Two major risk factors are overweight and age. We present objective and subjective cure rates of elderly and overweight patients treated with an adjustable single-incision sling system (AJUST™, C.R. BARD, Inc.).

**Materials and Methods:**

Between 04/2009 and 02/2012 we treated 100 female patients with the single incision sling. Patients were retrospectively evaluated by Stamey degree of incontinence, cough test, pad use, and overall satisfaction. The primary outcomes of the study were objective and subjective cure rates, secondary outcomes were the safety profile of the sling and complications.

**Results:**

The overall success rate in this population was 84.6% with a mean follow-up of 9.3 months. The average usage of pads per day decreased from 4.9 to 1.6 and was significantly lower in patients with a BMI <30 (p=0.004). Postoperative residual SUI was also lower in patients with a BMI <30 (p=0.006). Postoperative satisfaction was better in patients with a lower BMI, but this difference did not reach a level of significance (p=0.055). There were no complications such as bleeding, bladder injury, or tape infection.

**Conclusions:**

In elderly and obese patients a considerable success rate is achievable with this quick and minimal invasive procedure. However, the success rate shows a clear trend in favor of a lower body-mass-index. The cut-off point has been identified at a BMI of 30. The AJUST™ system can be regarded as safe and beneficial for elderly and obese patients.

## INTRODUCTION

Stress urinary incontinence (SUI) is the involuntary loss of urine during coughing or physical activity, mostly due to a weak pelvic floor or urethral sphincter (1). SUI derogates social, physical, psychological, occupational, and sexual aspects of life (2). Worldwide median prevalence of female urinary incontinence is 27.6%, divided in stress (50%), mixed (32%), and urge incontinence (14%) (3). With the increasing number of elderly people in the developed countries the prevalence of SUI is growing (4). It is estimated that the number of women with urinary incontinence in the USA will increase by 55% until 2050, thus affecting one third of all American women (5). With better information and a decreasing fear to report such symptoms more and more women are seeking help. The annual number of continence operations increased by 28% between 1997 and 2007 in the UK (6). More than 200.000 incontinence procedures per year are currently performed in the USA (7). The annual costs of stress urinary incontinence management are estimated to be $19.5 billion in the United States and ￡740 million in the United Kingdom (8).

Obesity and older age are two major risk factors of SUI. Furthermore parity, prior surgical treatment of the pelvic floor, menopausal status, smoking, coffee and alcohol consumption, and several concomitant diseases, e.g. COPD, chronic pelvic pain and constipation were identified as independent risk factors (1). Especially obesity is a demographic challenge of the future and the incidence is increasing dramatically. Between 1980 and 2008, mean global BMI increased by 0.4kg/m^2^ (men) and 0.5kg/m^2^ (women) per decade. In 2008 1.46 billion adults had a BMI ≥25 and about 0.5 billion ≥30 worldwide (9). The rising percentage of obesity is alarming, ans also WHO is concerned with the development of overweight people. BMI correlates with intra-abdominal pressure, potentially increasing the risk for SUI (10). Richter et al. found that obese patients undergoing SUI surgery complain of more incontinence episodes, more symptom distress, and worse quality of life (11).

With the growing number of elderly and overweight female patients with SUI a safe treatment procedure with little invasiveness, high efficacy, and good reproducibility is in great demand. Conventional tension free tapes show inferior results in older patients with a low pressure urethra and a higher morbidity in obese patients (12). Particularly the treatment of SUI in (mostly elderly) ISD (intrinsic sphincter deficiency) patients can mean a challenge. The TVT (tension free vaginal tape) procedure showed inferior results in Type III SUI compared to Type I/II SUI (13).

In our patient population we observed a high proportion of elderly and overweight females and therefore intended to review our results systematically concerning the outcome of an adjustable single incision sling (SIS) system (AJUST™, C.R.BARD Inc.) with particular focus on the correlation with obesity and age. The AJUST system offers adjustability of the tension applied to the urethra and minimal morbidity for no retrobubic or transobturator needle passage is necessary. Secondary outcomes included the safety profile of the sling device and possible complications, which are particularly important in an elderly population.

## MATERIAL AND METHODS

This is a retrospective analysis of 100 female patients with SUI treated between 04/2009 und 02/2012 with the adjustable single-incision sling system (AJUST™). All procedures were performed by the same surgeon (R.A.) with the experience of approximatly 400 tape procedures since the year 2000 including the first SIS (TVT Secur) in 2006.

The primary outcomes of the study were objective and subjective cure rates, evaluated by Stamey degree of incontinence (Grade 1: loss of urine with sudden increases of abdominal pressure: e.g. coughing, sneezing or laughing. Grade 2: loss of urine with lesser degrees of stress: e.g. walking or standing up. Grade 3: loss of urine without any relation to physical activity or position, e.g. while lying in bed.), cough test, pad use, and overall satisfaction. In accordance with the current literature and guidelines (21, 31) we do not systematically perform urodynamic testing in clearly demonstrable stress urinary incontinence on physical examination when it does not change management. Urodynamic testing was performed when other influencing factors were present, e.g. prior failed SUI surgery or neurological diseases. Postoperative evaluations were performed through clinical examination (60 patients) and telephone interview in all but one patient (1 to 4 times) to obtain subjective outcome. Patients were asked for continence status, pad use, urge symptoms, side effects (pain, bleeding), and overall satisfaction. The mean follow-up period was 9.3 months (1 to 23 months). The study protocol was approved by the local ethics committee (vote number 286/16).

**Figure 1:**
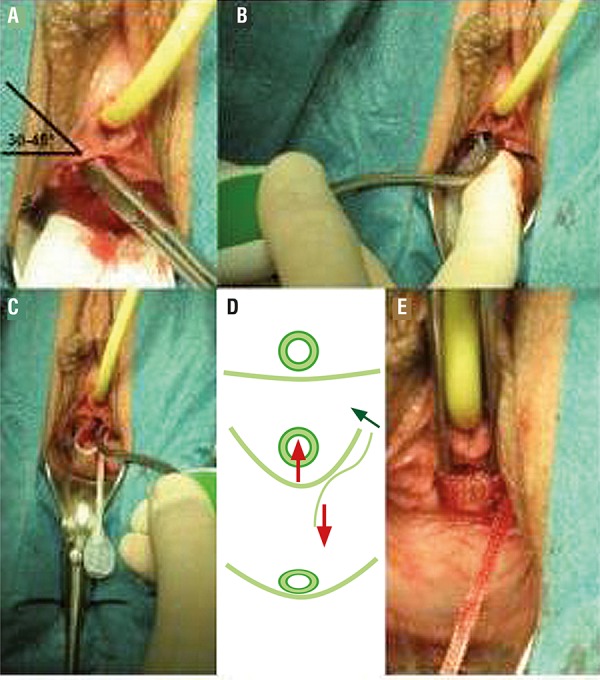
A) A bilateral small tunnel towards the inner margin of the inferior ramus pubis is created; B) Firstly the introducer is gently forwarded through the tunnel until the right obturator membrane is fully perforated (2 steps of resistance); C) The contralateral obturator membrane is perforated. The introducer shape is helpful in preventing a too deep insertion; D, E) A gentle tightening of the sling follows until no further leakage occurs through the Valsalva manoeuvre with 300cc bladder filling.

**Procedure (Figure-1):** A small incision underneath the mid-urethra is performed, the length must not be little more than 1cm what matches the width of the tape. This prevents dislocation of the tape during tensioning and in the postoperative period. A bilateral small tunnel towards the inner margin of the inferior ramus pubis is created. The introducer is gently forwarded through the tunnel under digital control until the right obturator membrane is fully perforated in the medial corner (2 steps of resistance related to the obturator fascia). Then, the contralateral obturator membrane is perforated. The introducer shape is helpful in preventing a too deep insertion, digital control prevents perforation of the vaginal skin in the sulcus. There follows a gentle tightening of the sling by pulling on the adjustment mesh arm until no further leakage occurs during the Valsalva manoeuvre with 300cc bladder filling. Under general or spinal anesthesia this is done through a Credé manoeuvre to create an intravesical pressure that approximately correlates with the cough pressure in an upright position. Special attention is paid to the prevention of overcorrection. If there is still some dribbling under adequate tension this is rather accepted than pulling too hard on the adjustment mesh what might end in extraction of the anchor and disruption of the obturator membrane. After adjustment a 14Fr. catheter must still smoothly pass the urethra. Certainly this testing adds to the operating time.

### Statistical analysis

Numerical variables are presented with mean and standard deviations. Comparative analysis of these variables were made with the Student`s t-test. Nominal or ordinale variables are presented as relative values. Chi-square and Fisher`s exact test were used to compare these statistics. Significance of all p-values was achieved at 0.05 in a two-tailed test. All statistical analysis were performed with SPSS statistical software (version 21.0).

## RESULTS

**Figure 2:**
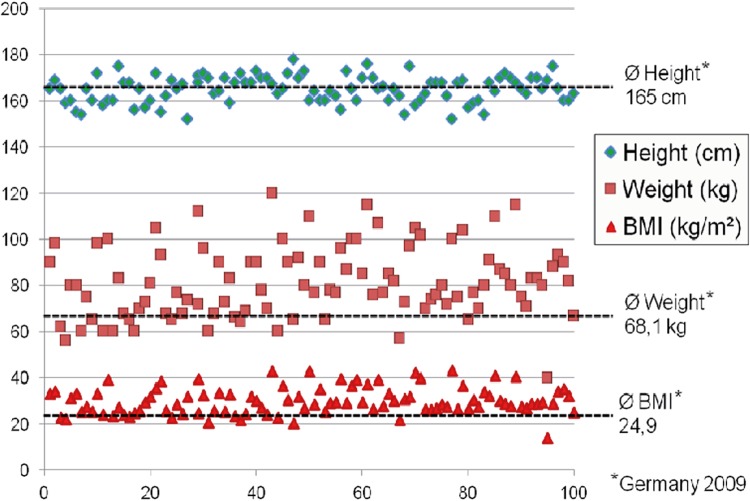
Age ranged from 43 to 86 years (mean 70.3 years). The body-mass-index (BMI) of the patients is predominantly clear above the German average.

Mean age of the patients was 70.3±8.7 years, mean parity was 2.5±1.7. 16/100 patients presented with I SUI according to the Stamey classification, 43/100 with II SUI, 18/100 with III SUI, and 23/100 with mixed incontinence and a predominant stress component (Table-1). Mean body-mass-index (BMI) was 28.7, 29.8, 30.9, and 30.2, for I, II, III SUI, and mixed incontinence, respectively, compared to an average BMI of 24.9kg/m^2^ in Germany 2009 (Figure-2). 88/100 patients had prior pelvic floor surgery (mean 2.2 (0-13) operations). The patients stayed in hospital for 3.1±1.8 days as usual in the German health system where these procedures are typically not performed as 1-day-surgery due to patient safety and reimbursement aspects (Table-1). Nearly all procedures were done under general or spinal anesthesia, only few patients opted for local anesthesia. No major concomitant procedures were performed, in 12 cases a tape resection or urethrolysis due to prior failed tape insertions was done, in 1 case a posterior mesh for rectocele correction was inserted at the same time. The procedures lasted 24.9±13.1 min on average and were easily feasible in all patients despite considerable periurethral scarring in many patients with prior interventions (Table-1). There were no complications like bleeding, bladder injury, or tape infection that required any kind of treatment. However, in 11 cases we observed urinary retention due to a suspected hypocontractile detrusor muscle, 7 temporary and 4 persistent. One of the latter was treated with a suprapubic catheter, three had a sling transection after 4, 5, and 11 months, respectively. Two of the latter remained completely dry, one re-developed first degree stress urinary incontinence.

**Table 1:** Pre-, intra-and postoperative data. Postoperative SUI and postoperative pad use are significant lower in patients with BMI <30. Data presented as mean value±standard deviation or relative frequency.

	All patients	BMI < 30	BMI ≥ 30	p
**Preoperative data**				
Parity	2.5 ± 1.7	2.3 ± 1.5	2.7 ± 1.9	0.297
Patient age [a]	70.3 ± 8.7	71.8 ± 7.8	68.3 ± 9.4	0.055
**Intraoperative data**				
Operation time [min]	24.9 ± 13.1	23.8 ± 11.8	26.3 ± 14.7	0.352
**Postoperative data**				
Hospital stay [d]	3.1 ± 1.8	3.3 ± 2.2	2.9 ± 1.0	0.284
Urge	20/99	12/56	8/43	0,804
SUI				0.006
- no	50/71	35/41	15/30	
- I°	10/71	3/41	7/30	
- II°	8/71	3/41	5/30	
- III°	3/71	0	3/30	
Positive stress test	14/91	5/54	9/37	0.075
Satisfaction				0.055
- very good	33/100	24/56	9/44	
- good	30/100	17/56	13/44	
- fair	11/100	5/56	6/44	
- poor	26/100	10/56	16/44	
Pad use	1.6 ± 2.3	1.3 ± 2.2	2.8 ± 2.9	0.004

**Figure 3:**
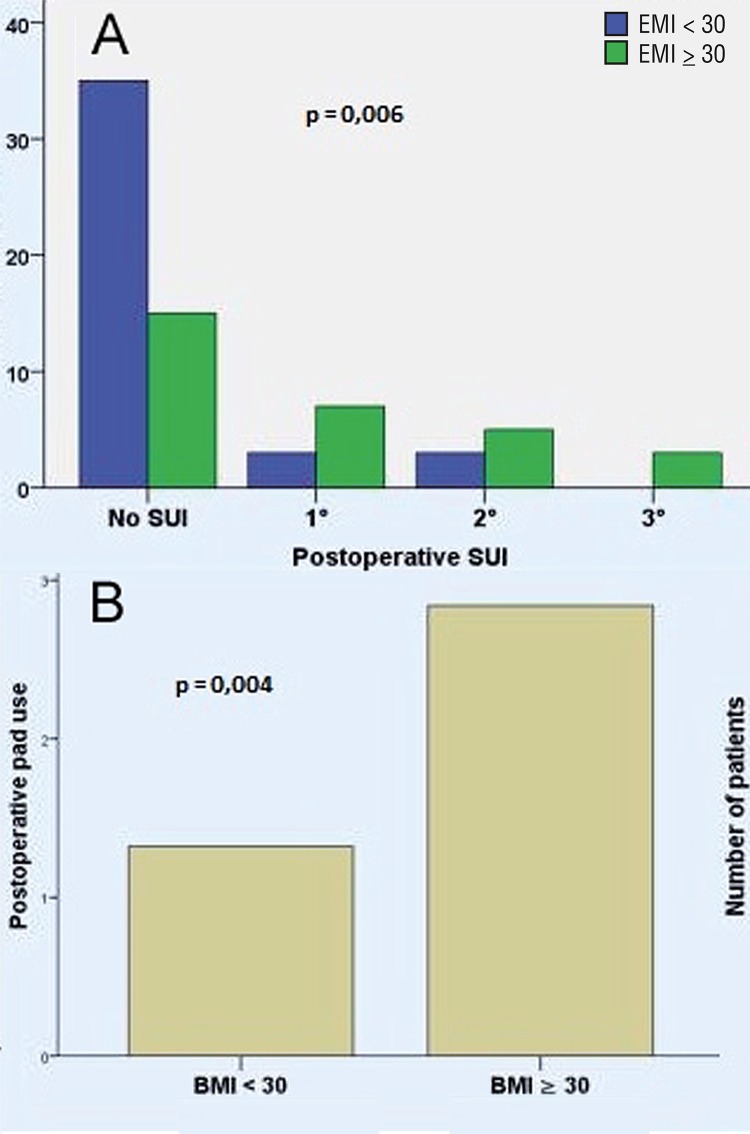
A) Postoperative SUI is significant lower in patients with BMI <30 (p=0.006); B) Postoperative pad use is significant lower in patients with BMI <30 (p=0.004).

77 of 91 clinically evaluable patients (84.6%) had a negative postoperative stress test what we considered objective cure. 9 patients were not available for postoperative reexamination for various reasons. We observed a distinct correlation with the BMI value: if BMI was <30 the pad test was negative in 90.7%. If BMI was >30 it was negative in 75.7%. The Stamey degree of SUI after the procedure was 0° in 50/99 (50.5%), I in 10/99 (10.1%), II 8/99 (8.1%) and III 3/99 (3.0%). 8/99 patients (8.1%) presented with mixed incontinence, 20/99 (20.2%) with isolated urge incontinence, 1 patient was not available for analysis due to language difficulties. 10/99 (10.1%) patients developed de novo urge symptoms, 6 of those with incontinence that was linked to poor satisfaction. 4 patients with de novo urge symptoms but without incontinence reported good or very good satisfaction. Comparative analysis showed a significantly better degree of SUI in patients with a BMI <30 (p=0.006, Figure-3a). No significant differences between patients with BMI <30 and patients with BMI ≥30 could be detected in pre-and intraoperative demographic data. Similarly, no significant difference between these two groups were found in postoperative length of stay and postoperative frequency of urge symptoms (p=0.804). The average usage of pads per day decreased from 4.9 (1-13) to 1.6 (0-10) and was significantly lower in patients with a BMI <30 (1.3±2.2 vs. 2.8±2.9; p=0.004, Figure-3b). 65/100 patients had a pad reduction of at least 50%.

**Figure 4:**
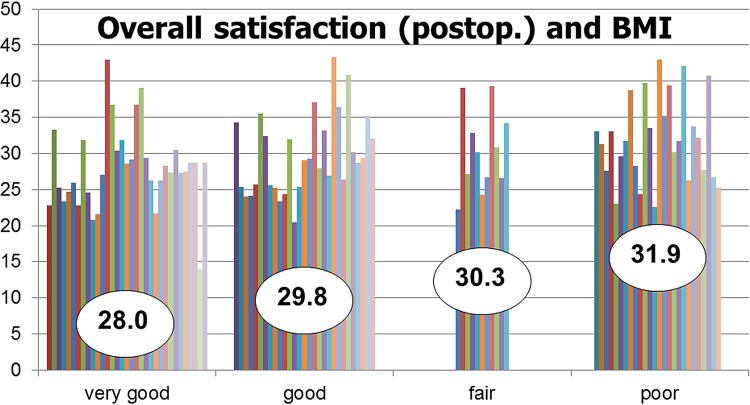
The average BMI of the patients with a very good, good, fair, and poor result was 28.0, 29.8, 30.3, and 31.9, respectively. A clear trend in favor of a lower BMI is obvious.

Overall satisfaction with the result, based on a subjective assessment of the patients was very good in 33/100 patients, good in 30/100, fair in 11/100, and poor in 26/100. The average BMI of the patients with a very good, good, fair, and poor result was 28.0, 29.8, 30.3, and 31.9, respectively (Figure-4). In general, postoperative satisfaction was better in patients with a lower BMI, but this difference did not quite reach a level of significance (p=0.055). 4 of the 26 patients who were regarded as failure underwent a surgical revision, 2 with sling shortening, 2 with a different sling.

## DISCUSSION

The proper treatment of stress urinary incontinence is continuously under debate. With the high number of procedures performed today, a highly competitive market for different tape implants has evolved. The now available longer-term data of implants have to compete with proven standard procedures. Jonsson et al. investigated long-term outcomes after different types of SUI surgeries. The Burch procedure had the lowest incidence of repeat SUI surgery (10.8%), followed by slings (13.0%), needle suspension (22.2%), and bulking agents (61.2%) (14). Novara et al. found similar results in a systematic review and meta-analysis (15). They reported higher objective continence rates using midurethral retropubic tapes compared to Burch colposuspension (odds ratio [OR]=0.38, p <0.0001), but with a high risk of bladder perforation (OR=4.94, p=0.00003). Midurethral tapes and pubovaginal slings showed similar results. Retropubic slings went along with slightly higher objective cure rates compared to transobturator tapes (TOT) (OR=0.8, p=0.04), but subjective cure rates were similar. Retropubic tapes had a higher risk of bladder perforation (OR=2.5, p <0.0001). With regard to the increasingly overweight population Subak et al. demonstrated a reduced frequency of self-reported urinary incontinence episodes in obese women after an intensive weight-loss program (16). Apparently the willingness for life style changes to reduce weight is commonly low, therefore treatment is requested.

Postoperative results of SUI surgery in obese patients are rated differently. The TOT procedure, for instance, is regarded as safe and successful. It avoids the retropubic space and causes fewer outflow obstructions (17). Frohme et al. did not find any influence of the BMI value on the outcome of TOT (18). Similarly Zivkovic et al. did not find any outcome differences between normal and overweight patients undergoing anterior colporrhaphy, anterior colporrhaphy with needle suspension of the bladder neck, and Burch colposuspension (19). Mukherjee et al. reported an equal effectiveness of TVT in obese women compared to lower BMI rates (20).

The current German interdisciplinary guidelines accept the use of single-incision slings due to the benefits of less invasiveness, less blood loss, and less postoperative pain compared to traditional slings. Complications with the blind TVT passage through the retropubic space (bladder, bowel, vascular injuries) can be avoided. Also injuries of the obturator nerve branches or groin pain caused by lesions of the adductor muscles by transobturator slings do not occur (15, 21-25).

In short term studies the outcome results of single incision slings are similar to retropubic or transobturator slings. Barber et al. found similar subjective cure rates after the prototype of mini-slings (TVT Secur, J&J Gynecare) or TVT after one year, (25) but this device failed to proof efficacy in later studies. In a meta-analysis by Abdel-Fattah et al. single-incision slings were inferior to standard midurethral slings concerning patient reported outcomes and objective cure rates and also showed higher reoperation rates (26). Similar results were reported by Djehdian et al., but noninferiority of the single-incision sling compared to a transobturator midurethral sling could not be demonstrated (27). These studies all include the learning-curve with SIS, longer term results considerably exceeding two years are still missing.

The adequate fixation by means of the anchoring systems is the crucial point of single-incision slings (28). Inferior results in the early studies can obviously be attributed to the immature technique of the TVT Secur that was not yet provided with a barbed hook (22). Such a barb was introduced in the Miniarc sling (AmericanMedicalSystems Inc.) that is designed to enter the obturator membrane, but not to fully perforate it. The adjustable single-incision sling system (AJUST™) provides a different anchoring system that is secured beyond the obturator membrane. Adjustment of the sling tension is made possible by a variable anchor after insertion. Midterm results after two years are promising and continence rates of 82.4% are comparable to TVT/TOT results, with fewer complications and significant improvements in quality of life indeces (23). In rare cases vaginal bleeding and erosion can occur (29, 30). Mostafa et al. described postoperative urinary retentions with a need for catheterization in 4.3% of patients (30). Meschia et al. mentioned 1/102 patients with postoperative urinary retention, resulting in tape resection (29). Naumann et al. described postoperative de novo urgency after AJUST™ insertion in 7.8% of patients. Only 1/52 patients complained of groin pain (23).

Our series demonstrates that the use of the adjustable single incision sling is particularly beneficial for obese patients as it avoids the passage through the retropubic and groin area. The design of the instrument allows a secure tape placement as obesity has usually no influence on vaginal anatomy. The minimal invasiveness allows the gentle treatment of elderly or frail elderly patients that are bothered by SUI. The crucial point is that both obese and older patients benefit from the adjustability of the tape. Only a careful and controlled degree of tension can make the difference for this ‘risk group’ of patients, especially when a low pressure urethra is present (Type III SUI) and the tension free principle has its limitations.

There is an ongoing debate if this condition has to be confirmed by urodynamic testing (UDS), but for straight forward SUI there is now consensus that UDS is not necessary as it has no impact on the outcome of surgery (31). Accordingly success rates in SUI studies are more defined by clinical parameters as mentioned above (32). In this context ample experience and a flair for the adequate tentioning is necessary, in particular with these apparently small procedures. The adjustment is supported by a ‘stress test’ with a filled bladder, an intraoperative cough test is mostly insufficient when the patient is under sedation and in a supine position. With an increasing degree of tension the risk of erosion or loosening of the anchors might also increase. Such complications did not occur in our series, but longer term studies should also focus on this issue.

In this regard we consider an objective cure rate of 84.6% and a subjective cure rate of 74% as reasonable in a group of the patients that might otherwise only be treated with absorbents. A 10% difference between objective and subjective cure rate is a well-known phenomenon in this field. Only a complete success meets the expectations of many patients despite all unfavorable conditions.

The retrospective design and the incomplete postoperative clinical examinations are the major limitations of the study. Nevertheless, this observational study obviously reflects real-life challenges of the health system with changing demographic parameters, in particular age and body weight. Ultimately, patient satisfaction is of paramount importance in SUI treatment.

## CONCLUSIONS

In our single-institution, single-surgeon experience the success rate shows a clear trend in favor of a lower body-mass-index. The cut-off point has been identified at a BMI of 30. But even in obese and morbidly obese patients a considerable success rate is achievable with this quick and minimal invasive procedure. The procedure can be regarded as safe as no complications were observed in this elderly group of patients in part with multiple risk factors. The individual adjustability of the sling in order to achieve an adequate degree of tension to the urethra is the key factor in restoring continence in this selected cohort of elderly and overweight patients. Further prospective studies comparing different types of sling systems are necessary to define the best approach for elderly and overweight patients.

## ABBREVIATIONS

BMI: Body Mass Index (kg/m)
SUI: Stress Urinary Incontinence
UK: United Kingdom
USA: United States of America
COPD: Chronic Obstructive Pulmonary Disease
WHO: World Health Organisation
ISD: Intrinsic Sphincter Deficiency
TVT: Tension free Vaginal Tape
SIS: Single Incision Sling
Fr.: French (1/3mm)
OR: Odds Ratio
TOT: Transobturator Tape
UDS: Urodynamics
